# *Klebsiella pneumoniae* and Colistin Susceptibility Testing: Performance Evaluation for Broth Microdilution, Agar Dilution and Minimum Inhibitory Concentration Test Strips and Impact of the “Skipped Well” Phenomenon

**DOI:** 10.3390/diagnostics11122352

**Published:** 2021-12-14

**Authors:** Rita Elias, José Melo-Cristino, Luís Lito, Margarida Pinto, Luísa Gonçalves, Susana Campino, Taane G. Clark, Aida Duarte, João Perdigão

**Affiliations:** 1 Research Institute for Medicines (iMed.ULisboa), Faculdade de Farmácia, Universidade de Lisboa, 1649-003 Lisboa, Portugal; rita.elias@tecnico.ulisboa.pt; 2 Laboratório de Microbiologia, Serviço de Patologia Clínica, Centro Hospitalar Universitário Lisboa Norte, 1649-049 Lisboa, Portugal; melo_cristino@medicina.ulisboa.pt (J.M.-C.); lmlito@chln.min-saude.pt (L.L.); 3 Instituto de Microbiologia, Faculdade de Medicina, Universidade de Lisboa, 1640-028 Lisboa, Portugal; 4 Laboratório de Microbiologia, Serviço de Patologia Clínica, Centro Hospitalar Universitário Lisboa Central, 1169-050 Lisboa, Portugal; margaridafeijopinto@gmail.com; 5 Serviço de Patologia Clínica, Hospital SAMS, 1849-019 Lisboa, Portugal; mariallgodinho@gmail.com; 6 London School of Hygiene and Tropical Medicine, London WC1E 7HT, UK; susana.campino@lshtm.ac.uk (S.C.); Taane.Clark@lshtm.ac.uk (T.G.C.); 7 Faculdade de Farmácia, Universidade de Lisboa, 1649-003 Lisboa, Portugal; 8 Centro de Investigação Interdisciplinar Egas Moniz, Instituto Universitário Egas Moniz, Quinta da Granja, 2829-511 Monte da Caparica, Portugal

**Keywords:** *Klebsiella pneumoniae*, colistin, susceptibility testing, skipped wells

## Abstract

The emergence of multidrug resistant Gram-negative pathogens, particularly carbapenemase producers, has forced clinicians to use last line antibiotics, such as colistin. Since colistin susceptibility testing presents several challenges, this study aimed at evaluating the performance of two alternative susceptibility methods for *Klebsiella pneumoniae*, namely, agar dilution (AD) and MIC test strips (MTS). These approaches were compared with the reference method, broth microdilution (BMD), and provide a quantitative description for the “skipped well” (SW) phenomenon. Colistin susceptibility was evaluated by BMD and AD in parallel and triplicate, using 141 *K. pneumoniae* clinical isolates while MTS performance was evaluated only for a subset (*n* = 121). Minimum inhibitory concentration analysis revealed that a substantial part (*n* = 26/141; 18.4%) of the initial isolates was deemed undetermined by BMD due to the following: discordance between replicates (1.4%); presence of multiple SWs (7.8%); and the combination of both events (9.2%). Both AD and MTS revealed a high number of false-susceptible strains (“very major errors”), 37.5% and 68.8%, respectively. However, AD agreement indices were reasonably high (EA = 71.3% and CA = 94.8%). For MTS these indices were lower, in particular EA (EA = 41.7% and CA = 89.6), but the approach enabled the detection of distinct sub-populations for four isolates. In conclusion, this study provides the most comprehensive study on the performance of AD and MTS for colistin susceptibility testing in *K. pneumoniae*, highlighting its limitations, and stressing the importance of sample size and composition. Further, this study highlights the impact of the SW phenomenon associated with the BMD method for *K. pneumoniae*.

## 1. Introduction

Colistin is a circular polycationic antimicrobial that has been regaining further attention as one of the last resort drugs against multidrug resistant (MDR) gram-negative pathogens, especially carbapenemase producers [1]. In more recent years, colistin resistance gained special importance due to the identification of plasmid mobilized resistance genes (*mcr*), presently comprising ten variants (*mcr*-1-10), and initially described in *Escherichia coli* and *Klebisella pneumoniae* isolates obtained from food-producing animals but subsequently detected across several gram-negative species [2,3]. Despite its increasing need and usage, reliable susceptibly testing to colistin still poses major challenges owing to the following: (i) the complex and multicomponent chemical structure of colistin [4]; (ii) cationic groups that enable the adherence to plastic surfaces [5]; (iii) its large molecular size; and (iv) the occurrence of phenomena such as the emergence of heteroresistance, persistence and/or tolerant subpopulations [6].

Currently, broth microdilution (BMD) is the gold standard method recommended by the EUCAST (European Committee on Antimicrobial Susceptibility Testing) and CLSI (Clinical and Laboratory Standard Institute) for colistin susceptibility testing. However, since BMD resorts to microtiter plastic plates and colistin has affinity to plastic, the results obtained by this method may be prone to reproducibility issues or inaccuracy [7,8]. Also, a distinct phenomenon commonly referred as “skipped wells” (SW), characterized by lack of growth in wells with intermediate colistin concentrations followed by growth in wells with higher concentration, has been observed and reported by some studies but never properly quantified for *Klebsiella pneumoniae* [9,10]. The underlying cause of this phenomenon is unknown and poses an additional challenge to the results produced by this method. It has been speculated that it may be derived from the plastic adherence problem, but this latter hypothesis would imply that this phenomenon should, in theory, manifest equally across different bacterial species. However, the existing data reported in the literature show that it mostly affects a restricted group of species, including *K. pneumoniae* [9,11].

Moreover, the BMD method is too laborious to be performed routinely and on a day-to-day patient care basis. Instead, most laboratories rely upon automated systems, diffusion methods such as disk diffusion and Minimum inhibitory concentration (MIC) Test Strips (MTS) or, molecular approaches. Unfortunately, diffusion methods are inadequate for colistin susceptibility testing due to its large molecular size, which compromises diffusion into the medium and thereby leads to results that may be unreliable [12,13]. For instance, Tan et al. (2006) investigated the response of 228 clinical isolates of *Acinetobacter* spp., *Pseudomonas aeruginosa* and Enterobacteriaceae spp. (of which 42 are *Klebsiella* spp.) to three different disk diffusion methods and all denoted high proportions (79.0–89.0%) of false-susceptible isolates [13]. Moreover, Rojas et al. (2017) showed that more than a third (35.5%) of the *K. pneumoniae* isolates were misclassified by MTS (Etest) as susceptible [14]. For molecular methods, the complex regulatory network of colistin resistance makes it unfeasible to screen all genes involved [15]. Several studies have explored different antimicrobial susceptibility testing methods for colistin, including automated systems (e.g., Vitek2 and BD Phoenix), in *K. pneumoniae* and other Enterobacteriaceae, but the results are difficult to compare. Since most of studies tend to aggregate their data for multiple species, it is unclear how these methods perform for each species [11,12,13,16,17]. Also very common is the absence of discriminated data for all the methods tested [18,19], the utilization of other methods than BMD as reference [13,20], the use of different indices to evaluate the performance of each method, which hampers the comparative analysis or further meta-analysis of available data [9] and, the use of small samples with low number of susceptible isolates [9,17,21].

Since BMD, agar dilution (AD) and MTS allow for the quantitative determination of colistin susceptibility and correlation with qualitative susceptibility determination, the present study evaluates the performance of AD and MTS susceptibility testing methods for colistin in *K. pneumoniae*. BMD is herein used as the gold-standard while also enabling a quantitative description of the SW phenomenon.

## 2. Materials and Methods

### 2.1. Experimental Framework and Bacterial Isolates

The study includes a diverse sample of 141 *K. pneumoniae* clinical isolates available from the strain collection of the Faculty of Pharmacy of the University of Lisbon. All isolates were obtained between 1980 and 2019 from different hospitals in Lisbon, Portugal, and were isolated from infected/colonized patients or hospital environmental sources (surfaces and objects) (Supplementary Table S1). Envisaging the comparison between different susceptibility methods, all isolates were tested for colistin susceptibility by BMD (reference method) and agar dilution, with colistin sulphate salt (ACROS organics, Morris Plains, NJ, USA; Lot A0372655) adjusted for its purity fraction. Also, colistin susceptibility testing by MTS (Etest) was carried out for 121 isolates. *Escherichia coli* ATCC 25922 (MIC = 0.25–2 μg/mL) and *Pseudomonas aeruginosa* ATCC 27853 (MIC = 0.5–4 μg/mL) were used as quality control strains as per recommendations of the CLSI [21].

### 2.2. Broth Microdilution

BMD was carried out in triplicate using untreated, polystyrene, 96-well plates (U-shaped, Greiner Bio-One, Frickenhausen, Germany) where colistin concentrations were obtained by serial two-fold dilutions on cation-adjusted Muller-Hinton broth (CAMHB, Oxoid, Basingstoke, UK) reaching a concentration range of 0.25–16 μg/mL. Bacterial suspensions were then inoculated, to each well as to achieve a final concentration of 10^5^ CFU/mL. To monitor the healthiness of isolates and media sterility, a drug-free line and a non-inoculated column were included in each assay. The plates were incubated overnight at 37 °C [8]. The lowest concentration inhibiting bacterial growth was considered the MIC value and the interpretation of these results was done in accordance with EUCAST breakpoints for Enterobacteriaceae [22]: isolates with a MIC ≤ 2 μg/mL were categorized as susceptible whereas those with a MIC > 2 μg/mL were classified as resistant (Supplementary Table S1).

### 2.3. Agar Dilution

MIC determination by AD was performed in triplicate on Mueller-Hinton Agar (MHA) plates supplemented with colistin sulphate in a concentration range of 0.25 to 16 μg/mL. Drug-free MHA plates were used as growth control. Through a multipoint inoculator, 52 equally spaced bacterial suspensions were inoculated to each plate as to reach a final concentration of 10^4^ CFU/spot. Plates were incubated overnight at 37 °C and the MIC values recorded were the lowest concentration inhibiting bacterial growth (Supplementary Table S1) [23].

### 2.4. MIC Test Strip (Etest)

For each isolate, a bacterial suspension was prepared and adjusted to a 0.5 MacFarland standard, spread on an MHA plate and a MIC test strip (HIMEDIA, Mumbai, India) with colistin concentrations of 0.016 to 256 μg/mL was added. Plates were incubated overnight at 37 °C and the MIC values recorded as per the manufacturer’s instructions (Supplementary Table S1).

### 2.5. Statistical Analysis and Performance Evaluation

The correlation between susceptibility methods was evaluated by the Pearson method with values ranging from −1 (perfect negative correlation) to +1 (perfect positive correlation) [24,25]. Also, another four indices were calculated: essential agreement (EA), categorical agreement (CA), major error (ME) and very major error (VME). EA and CA are related to reproducibility, EA is the percentage of isolates with the same MIC or with a variation of ±1 dilution between the two methods, and CA is the percentage of isolates with a concordant susceptible/resistant phenotype between the two methods. ME and VME calculate the error between methods, ME is the percentage of false-resistant in the total susceptible isolates obtained by BMD, and VME is the percentage of false-susceptibilities among the total resistant isolates obtained with BMD. Correlation and association results between susceptibility methods are considered good when the Pearson correlation score is closest to 1, CA ≥ 90%, EA ≥ 90%, ME < 3% and VME < 3% (Table 1) [26,27]. When multiple isolates of a given species are being tested for susceptibility, MIC_50_ and MIC_90_ are two important parameters to report, representing the MIC values at which ≥50% and ≥90%, respectively, of the isolates in a test population are inhibited [28]. Receiver operating characteristic (ROC) curves were obtained by IBM SPSS Statistics (v. 28, IBM, Armonk, NY, USA) and allow to compare the sensitivity and specificity between susceptibility testing methods. Sensitivity and specificity measure the capacity to identify the true resistant- and true susceptible-isolates, respectively. The accuracy of each technique can be provided by the area under the ROC curve (AUC) [29].

## 3. Results

### 3.1. Colistin MICs for Klebsiella pneumoniae as Determined by BMD and AD

**Figure 1:**
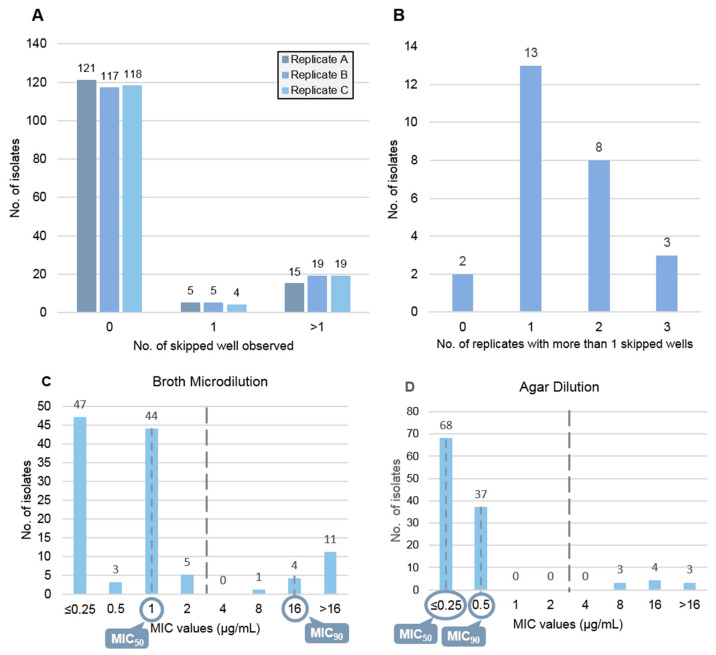
Results obtained for colistin susceptibility testing by BMD (reference method) and AD. (**A**) Number of isolates with 0, 1 or more than 1 SW in each of the three replicates tested (**A**–**C**) by BMD. (**B**) Distribution of isolates with undetermined MIC (*n* = 26) by the number of replicates showing more than one SWs: all replicates (*n* = 3), 2, 1 or none (*n* = 0). MIC distribution obtained by (**C**) broth microdilution and (**D**) agar dilution, MIC_50_ and MIC_90_ values are highlighted for each method.

This study includes a total of 141 *K. pneumoniae* clinical isolates for which colistin susceptibility was evaluated in vitro by BMD and AD with both methods carried out in parallel and in triplicate (Replicates A, B and C). Results produced by BMD revealed reproducibility issues, with disagreements between replicates, which were in part related with SW events. Between 15–19 isolates in each replicate exhibited more than one SW: 15 isolates (11%) in replicate A, 19 (13%) in replicate B and 19 (13%) in replicate C. Single SW events, considered as valid, were also observed but at lower frequencies between replicates: 5 isolates (4%) in replicate A, 5 (4%) in replicate B and 4 (3%) in replicate C (Figure 1A). These reproducibility issues hampered colistin MIC determination for 26 (18.9%) isolates, 3 of which with more than 1 SW in all replicates, 8 showed more than 1 SWs across 2 replicates, 13 had more than 1 SWs in 1 replicate and 2 did not showed more than 1 SWs (Figure 1B). Among the latter two isolates, none of the MICs obtained in the three replicates were discrepant by more than one-fold dilution range. Therefore, the reason behind indetermination of the 26 colistin MICs were the following: (i) multiple SW (*n* = 11); (ii) disagreement between at least two replicates (*n* = 2); and (iii) the combination of both, multiple SW, and lack of agreement between the remaining replicates (*n* = 13). (Figure 1B).

Upon exclusion of isolates with indeterminate MICs, MIC data obtained for 115 (81.6%) isolates was therefore retained for comparative analysis and performance evaluation. For this sample, BMD MIC results varied from ≤0.25 to >16.0 μg/mL with 99 susceptible (MIC ≤ 2 μg/mL) and 16 resistant isolates (MIC > 2 μg/mL). The distribution of MIC results obtained with BMD showed clearly divergent MIC_50_ and MIC_90_ values of 1 μg/mL and 16.0 μg/mL, respectively (Figure 1C).

Regarding susceptibility testing by AD, MIC values ranged between ≤0.25 and >16.0 μg/mL, with 131 susceptible (MIC ≤ 2 μg/mL) and 10 resistant isolates (MIC > 2 μg/mL) (Supplementary Table S1). Upon comparison across the 115 isolates with BMD-based MIC data, the MIC distribution of AD (*n* = 115; 105 susceptible- and 10 resistant- isolates) was markedly different from BMD, as well as the MIC_50_ and MIC_90_ values, ≤0.25 μg/mL and 0.5 μg/mL, respectively (Figure 1D). MICs of quality control strains were in accordance with the established values for the two methods.

### 3.2. Comparing Performance of Agar Dilution for Colistin Susceptibility Testing with Broth Microdilution

**Figure 2:**
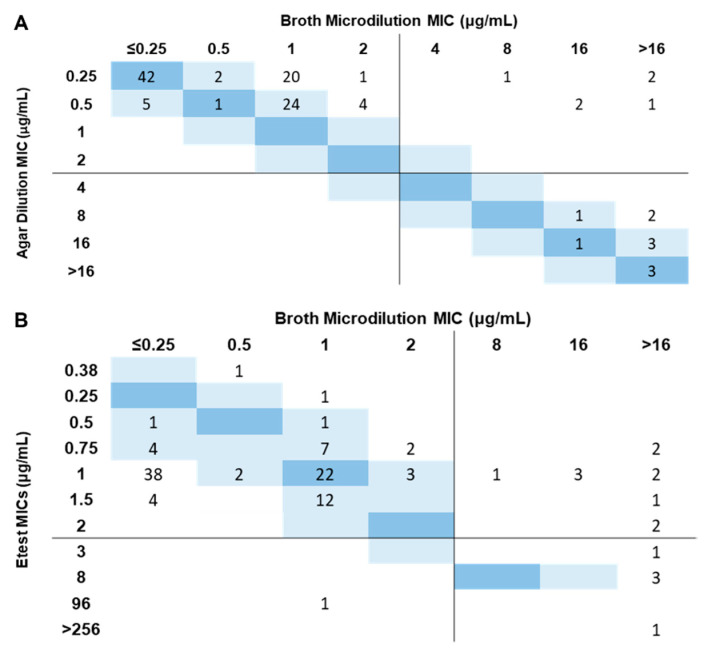
Correlation between the reference method, broth microdilution, and AD (**A**) or MTS (**B**). Blue rectangles represent the number of isolates within the MIC essential agreement range where the darker ones represent the number of isolates with the same MIC and the light ones the number of isolates within ±1 two-fold change. The black lines separate the susceptible (≤2 μg/mL) and resistant (>2 μg/mL) phenotypes according to EUCAST breakpoints [22].

MIC distribution was markedly different between methods as AD did not produce intermediate MICs (1–4 μg/mL) and 1 μg/mL was the second most common MIC value recorded by BMD (Figure 1C,D). Categorical Agreement and Essential Agreement were shown to be above and below the acceptable value of ≥90%, respectively, with CA equal to 94.8% and EA equal to 71.3%. Regarding the error indices, ME rate associated with AD was found to be 0% whereas the AD very major error (VME) rate was determined to be 37.5% (Table 1). Moreover, the correlation between reference MICs and those obtained with AD (Figure 2A) was deemed as poor (Pearson’s r = 0.5).

**Figure 3:**
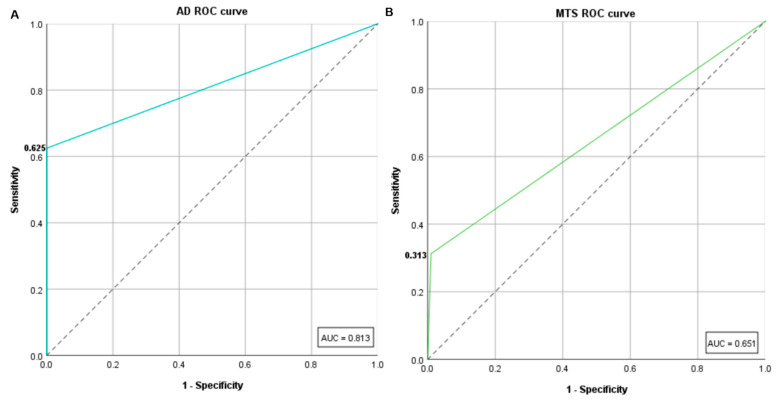
Receiver operating characteristic (ROC) curves of AD (**A**) and MTS (**B**) and their respective areas under the ROC curves (AUC), 0.8 and 0.7.

To evaluate the performance of AD, sensitivity and specificity were calculated. AD identified correctly 10 out of the 16 resistant isolated classed by BMD, resulting in a sensitivity of 62.5%. On the other hand, the capacity of AD to identify true susceptible isolates was higher, with no isolate erroneously classed as resistant (*n* = 99/99; 100%). The calculated area under the ROC curve (AUC) for AD was 0.8 (Figure 3A).

### 3.3. MTS Is Associated with High VME Rates but Enables the Detection of Distinct Sub-Populations

With the objective of not only comparing the performance of MTS but also to screen for heteroresistance, MTS was performed for the 115 *K. pneumoniae* isolates for which concordant MIC values were obtained by BMD. MTS MIC results ranged between 0.38 and >256.0 μg/mL, with the majority (*n* = 74/115; 64.3%) falling into 1 μg/mL, including the quality control strains (Figure 2B and Supplementary Table S1). MTS data denoted a high error rate with 11 false-susceptible isolates out of 16 resistant isolates (VME = 68.8%) whereas only one false-resistant was identified (ME = 1.0%), which is reflected in a specificity of 99.0% (Figure 2B and Table 1). Essential agreement (EA 41.7%), Person’s correlation coefficient (r = 0.3) and sensitivity of MTS (31.3%) were low with a ROC AUC of 0.7, lower than the ROC AUC obtained by AD. Categorical agreement (CA 89.6%) was found to be marginally below the acceptance criterium (<90) (Figure 3B and Table 1).

Nonetheless, MTS revealed two distinct populations (with different MICs) for four isolates, three of which were considered resistant to colistin by BMD (Kp4129, MIC = 8 μg/mL; Kp5509 and Kp5514 with MIC > 16 μg/mL) and one susceptible (Kp4857, MIC = 1 μg/mL) (Supplementary Figure S1). For isolates Kp4129 and Kp5514, a susceptible population with MIC of 1 μg/mL and a resistant one with MIC of 4 and 3 μg/mL, respectively, are noticeable (Supplementary Figure S1A,G). The presence of two sub-populations is even more evident in KP4857 and Kp5509 with MIC discrepancies from 0.75 μg/mL to 96 μg/mL and 12 μg/mL to >256 μg/mL, respectively (Supplementary Figure S1C,E). MTS was then repeated for these isolates but only using the highest MIC sub-populations: the same pattern was again observed for Kp5514 and Kp5509 (with two sub-populations), although in Kp5509, a more uniform growth with a resistance level of >256 μg/mL was obtained (Supplementary Figure S1F,H). On the other hand, for Kp4129 and Kp4857, higher resistance levels of 6 and >256 μg/mL, respectively, were observed (Supplementary Figure S1B,D). The diversity and phenotypic heterogeneity herein observed supports the existence of additional mechanisms involving transiently induced phenotypic resistance and/or heteroresistance whereby different subpopulations comprise the clinical isolate.

## 4. Discussion

BMD is considered the gold-standard for colistin susceptibility testing, but it suffers from several issues already outlined that affect its performance. Yet, as colistin is being increasingly used as a last treatment option the importance of obtaining reliable susceptibility test results for this drug is paramount. Since alternative methods are available but have not been exhaustively evaluated for *K. pneumoniae*, we sought to evaluate the performance of AD and MTS and compare these to BMD on a set of 141 *K. pneumoniae* isolates obtained over approximately four decades. This unique collection enabled us to include isolates subjected to distinct antibiotic selective pressures. Also, by performing BMD and AD in triplicate we were able to quantify the reproducibility of these methods.

We observed that discordance between two or more replicates coupled with several SW events hampered the determination of colistin MIC of 18% (*n* = 26/141) of the isolates included in this study. This subset comprises a substantial proportion of the study sample and for which the MIC was undetermined due to either discordance (*n* = 2, 7.7%), the presence of multiple SWs (*n* = 11, 42.3%), or both events (*n* = 13, 0.5%). In fact, this study observed multiple and single SWs in 18% and 5% of each replicate, respectively. While the SW phenomenon has been previously reported [9,10,14], to our knowledge this is the first time it is quantified for *K. pneumoniae* using not only multiple replicates but also a larger study sample. Matuschek et al. (2017) observed this phenomenon but since they retested the strains until obtaining a MIC value, it is not possible to quantify the impact of this phenomenon [10]. Turlej-Rogacka et al. (2018) also reported SWs but only in one out of four *K. pneumoniae* [9].

Alternatively, AD for colistin susceptibility testing should, in theory, overcome the plastic adherence problem as well as those derived from the presence of SWs since the drug is incorporated into melted media. As we evaluated the performance of AD in relation to BMD, the major difference observed pertained with an AD tendency to underestimate the MIC values of some resistance strains, which is reflected in the number of false-susceptible strains (6/16; VME = 37.5%) and sensitivity of 62.5%, when considering BMD as the reference method. Apart from this, CA was high (94.8%) and AD specificity was very high (100%), but EA was slightly below the acceptance threshold as defined by the CLSI (71.3% < 90%). Similar values were already reported by Dafopoulou et al. (2015), in which AD classified all isolates (*n* = 41) as resistant, leading to a CA equal to 97.6%, and no ME (0.0%), but VME rate of 2.4%, all meeting the minimum acceptance values as per CLSI recommendations [27,28,30]. However, this latter study included only one *K. pneumoniae* susceptible isolate therefore imposing a limitation to the values these performance indices can assume, particularly VME. Turlej-Rogacka et al. (2018) also evaluated the performance of AD for colistin AST and results revealed high reproducibility for different replicates of the same plate, in either fresh or one-week storage plates but the sample was small (*n* = 4) [9]. By including 141 *K. pneumoniae* isolates, 16 of which were classified as colistin resistant by the gold standard method (BMD), the present study provides a more comprehensive and robust sample while stressing the importance of sample size and composition in the performance evaluation for a given susceptibility testing method. Herein, the importance of sample size and structure is particularly relevant in the uncovering of a high VME rate previously unreported for AD.

Regarding MTS, concordance between MTS and BMD was already expected to be poor in line with previous studies [13,31]. Herein, we report a high rate of false susceptible isolates (VME 68.8%), low essential and categorical agreements (EA 41.7%; CA 89.6%) and low sensitivity for the detection of colistin resistance (31.3%). The Dafopoulou et al. (2015) study mentioned above also evaluated the performance of MTS, and revealed similar results, with low agreement with BMD as reference (EA 48.8%; CA 56.1%) and a high rate of false susceptible isolates (VME 41.5%) [31]. MTS high VME rate and low EA are comparable to the ones obtained by the AD method, but both are above and below the recommended thresholds, respectively.

As the alternative methods to BMD herein studied do not provide satisfactory performance, this study also stresses that new approaches and/or revised methodologies may therefore be necessary to overcome the problem of colistin adsorption to plastic surfaces. This is an already well described issue, known to affect the reproducibility of MIC determination and influenced by both the plastic polymer (either polystyrene or polypropylene) and surface treatment [30]. In fact, not only are the MICs of colistin and polymyxin B lower when determined on polypropylene plates and compared to polystyrene plates, as the use of tissue-culture-coated polystyrene plates is discouraged since these have been found to be associated with an overall 5.3-fold increase in colistin MICs [16]. The supplementation with surfactants, such as polysorbate 80, has been proposed by different studies highlighting its role in decreasing the MICs of colistin, in particular at lower concentration ranges (≤2 µg/mL) [32,33]. Nonetheless, as recently reviewed by Ezadi et al. (2019), polysorbate 80 can also display antibacterial activity and synergism with polymyxins and neither the CLSI or EUCAST currently recommend supplementation with polysorbate 80 for the determination of colistin or polymyxin MICs [5]. In this regard, glass-coated plates and nonspecific binding plates have been reported as alternative vessels for MIC determination with the advantage of avoiding media supplementation with surfactants [34,35].

However, it is worth mentioning that MTS did allow the identification of four isolates, (Kp4129, Kp4857, Kp5509 and Kp5514), composed of at least two distinct subpopulations with the ability to survive increasing colistin concentrations. Kp4129, Kp4857 and even Kp5509 did in fact comprised a more resistant sub-population that when subjected to colistin susceptibility testing by MTS showed a higher MIC and no subpopulation and, is therefore strongly suggestive of heteroresistant isolates [6,36]. A distinct situation was found to be illustrated by Kp5514 since retesting by MTS reproduced the same pattern. This ability to survive under higher antibiotic concentrations without altering their MIC may be indicative of tolerance and/or persistence towards colistin [37]. Further studies are still warranted as to understand the mechanisms underpinning these phenomena including population analysis profiling (PAP) [38] and determination of MDK_99_ and MDK_99.9_ [39] as to further confirm these findings. Nevertheless, the MTS ability is relevant for identifying such situations that may otherwise be unnoticed and is in fact proposed by Halfawy and Valvano (2015) to be implemented as a first screening step for heterorresistance detection, followed by PAP [38].

## 5. Conclusions

In conclusion, to our knowledge this study provides the most comprehensive study on the performance of AD and MTS for colistin susceptibility testing in *K. pneumoniae*, highlighting its limitations, particularly the high VME rate which is transversal to both methods. Also, the study provides a quantitative description on specific issues such as the SW phenomenon and precision associated with the BMD method in *K. pneumoniae*. Since other species are prone to these same problems, and since colistin is gaining an increased importance at the last line of treatment, further studies focused on other bacterial species are warranted as to fully understand the existing limitations in colistin susceptibility testing and the need for further standardization of the methodology.

## Figures and Tables

**Table 1:** Agreement and error indices for agar dilution and MIC test strip methods in comparison with broth microdilution.

Indices	Formula	AD	MTS	Acceptance Criteria ^1^
Categorical Agreement (CA)	$\frac{N_{CA}}{NT}\times 100$	N_CA_ = 109 N_T_ = 115 **CA = 94.8%**	N_CA_ = 103 N_T_ = 115 **CA = 89.6%**	≥90%
Essential Agreement (EA)	$\frac{N_{EA}}{NT}\times 100$	N_EA_ = 82 N_T_ = 115 **EA = 71.3%**	N_EA_ = 48 N_T_ = 115 **EA = 41.7%**	≥90%
Major Error (ME)	$\frac{N_{ME}}{N_{RefS}}\times 100$	N_ME_ = 0 N_RefS_ = 99 **ME = 0%**	N_ME_ = 1 N_RefS_ = 99 **ME = 1.0%**	<3%
Very Major Error (VME)	$\frac{N_{VME}}{N_{RefR}}\times 100$	N_VME_ = 6 N_RefR_ = 16 **VME = 37.5%**	N_VME_ = 11 N_RefR_ = 16 **VME = 68.8%**	<3%

N_CA_—number of isolates with a MIC result within the same categorical interpretation as reference method; N_T_—total number of isolates tested; N_EA_—number of isolates with the same or ± 1 doubling dilution MIC value as the reference method; N_ME_—number of isolates that yielded false-resistant results; N_RefS_—number of isolates susceptible by the reference method; N_VME_—number of isolates that exhibit false-susceptible results and N_RefR_—number of isolates resistant by the reference method.^1^ CLSI, 2016 [26] and CLSI, 2016 [27].

## Supplementary Materials

The following are available online at https://www.mdpi.com/article/10.3390/diagnostics11122352/s1, Figure S1: Colistin MTS results of Kp4129 (A); Kp4129 resistant subpopulation (6 μg/mL) (B); Kp4857 (C); Kp4857 resistant subpopulation (>256 μg/mL) (D); Kp5509 (E); Kp5509 resistant subpopulation (>256 μg/mL) (F) Kp5514 (G); Kp5514 resistant subpopulation (H), Table S1: Features of *Klebsiella pneumoniae* isolates used in this article and colistin susceptibility results by broth microdilution, agar dilution and Minimum inhibitory concentration Test Strips (MTS).
