# Supplementary material for: Klebsiella pneumoniae and Colistin Susceptibility Testing: Performance Evaluation for Broth Microdilution, Agar Dilution and Minimum Inhibitory Concentration Test Strips and Impact of the “Skipped Well” Phenomenon
Source: Diagnostics (Basel). 2021 Dec 14;11(12):2352. doi: 10.3390/diagnostics11122352 (PMC8700027; doi:10.3390/diagnostics11122352)
Supplement: Supplementary file 1 [file diagnostics-11-02352-s001.zip › diagnostics-1474338-supplementary/Figure S1.pdf]

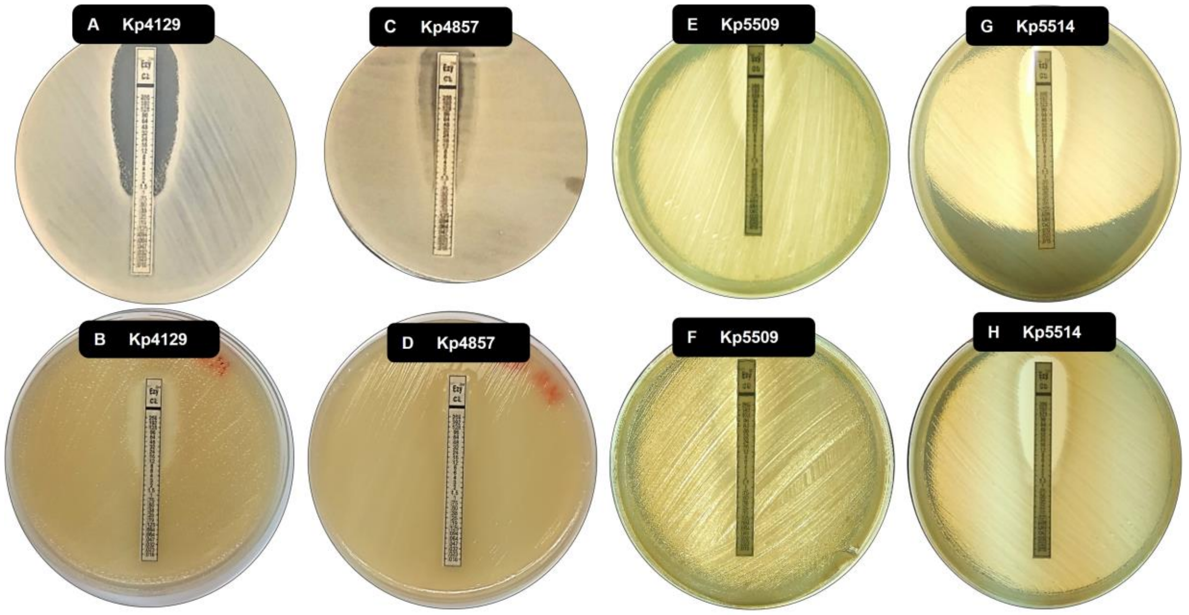

Figure S1: Colistin MTS results of Kp4129 (A); Kp4129 resistant subpopulation (6 g/mL) (B); Kp4857 (C); Kp4857 resistant subpopulation (>256 g/mL) (D); Kp5509 (E); Kp5509 resistant subpopulation (>256 g/mL) (F) Kp5514 (G); Kp5514 resistant subpopulation (H)
