# Supplementary material for: Klebsiella pneumoniae and Colistin Susceptibility Testing: Performance Evaluation for Broth Microdilution, Agar Dilution and Minimum Inhibitory Concentration Test Strips and Impact of the “Skipped Well” Phenomenon
Source: Diagnostics (Basel). 2021 Dec 14;11(12):2352. doi: 10.3390/diagnostics11122352 (PMC8700027; doi:10.3390/diagnostics11122352)
Supplement: Supplementary file 1 [file diagnostics-11-02352-s001.zip › diagnostics-1474338-supplementary/Suplementary Table S1.pdf]

Supplementary Table S1 - Features of *Klebsiella pneumoniae* isolates used in this article and colistin susceptibility results by broth microdilution, agar dilution and Minimum inhibitory concentration Test Strips (MTS).

Strain	ST	Year of Isolation	Environmental/ Biological source	Broth microdilution MIC (µg/mL) (Skipped wells)			Agar dilution MIC (µg/mL)			MTS MIC (µg/mL)
				Replica A	Replica B	Replica C	Replica A	Replica B	Replica C	
KP684	ST25	1999	Urine	0.5	0.25	0.25	0.5	0.5	0.25	1.0
KP4129	ST14	1980	Ear	8.0	8.0	4.0	0.25	0.25	0.25	1.0
KP4151	ST252	1980	Pharyngeal exudate	0.25	ND (0.25 and 1.0)	ND (0.25-2.0)	0.25	0.25	0.25	-
KP4164	ST20	1980	Bath	ND (1.0; 4.0 and 8.0)	>16.0	>16.0	0.25	0.25	0.25	0.75
KP4228	Unknown	1980	Urine	0.25	0.25	ND (0.5-4.0)	0.25	0.25	0.25	1.0
KP4246	ST15	1980	Urine	1.0 (0.25)	0.5	0.5	0.25	0.25	0.25	0.38
KP4247	ST15	1980	Urine	8.0(0.25)	0.25	ND (0.5-4.0)	0.25	0.25	0.25	-
KP4248	ST3	1980	Bronchial secreation	ND (0.5 and 1.0)	ND (0.5-2.0)	ND (1.0 and 2.0)	0.5	0.5	0.5	-
KP4254	ST15	1980	Urine	ND (0.25 and 0.5)	ND (0.25 and 1.0)	ND (0.5- 2.0)	0.25	0.25	0.25	-
KP4256	ST37	1980	Urine	0.25	0.25	0.25	0.25	0.5	0.25	1.5
KP4257	ST15	1980	Urine	>16.0	>16.0	16.0	0.25	0.25	0.25	0.75
KP4171	ST1728	1981	Belly button	ND (0.25 and 0.5)	ND (0.25; 1.0; 2.0 and 8.0)	1.06 (4.0)	0.25	0.25	0.25	-
KP4184	ST37	1981	Pharyngeal exudate	0.25	ND (0.25-4.0)	ND (0.25-1.0)	0.25	0.25	0.25	-
KP4212	ST15	1981	Brush	0.25	ND (0.5; 1.0)	0.5	0.25	0.25	0.25	-
KP4214	ST2493	1981	Bench	ND (0.25; 0.5 and 4.0)	ND (0.5 and 1.0)	0.25	0.25	0.25	2.0	-
KP4263	ST15	1981	Urine	ND (0.5-8.0)	2.0	4.0 (1.0)	0.5	0.5	0.5	-
KP4264	Unknown	1981	Urine	0.5	0.5	0.5	0.25	0.25	0.25	1.0
KP4265	Unknown	1981	Urine	0.5	0.25	0.5	0.5	0.5	0.5	1.0
KP4194	ST37	1982	Pharyngeal exudate	ND (0.25 and 0.5)	0.25	0.25	0.25	0.25	0.25	1.0
KP4195	ST15	1982	Feces	1.0	1.0	1.0	0.25	0.25	0.25	0.75

Supplementary Table S1 - *continued*

Strain	ST	Year of Isolation	Environmental/ Biological source	Broth microdilution MIC (µg/mL) (Skipped wells)			Agar dilution MIC (µg/mL)			MTS MIC (µg/mL)
				Replica A	Replica B	Replica C	Replica A	Replica B	Replica C	
KP4197	ST1799	1982	Feces	0.25	0.25	0.25	0.25	0.25	0.25	1.0
KP4279	ST13	1982	Catheter	0.25	0.25	0.25	0.25	0.25	0.25	1.5
KP4287	ST25	1982	-	0.25	0.25	0.5	0.25	0.25	0.25	1.0
KP4292	ST70	1995	Blood	0.5	0.25	0.25	0.25	0.25	0.25	1.0
KP4297	ST25	1995	Blood	0.5	ND (0.25-1.0)	0.25	0.5	0.5	0.5	-
KP4325	ST25	1995	Blood	ND (0.5 and 1.0)	0.5	ND (0.5 and 1.0)	0.25	0.25	0.25	-
KP4333	ST252	1995	Blood	0.25	ND (0.25; 1.0 and 2.0)	0.25	0.5	0.5	0.25	1.0
KP4367	ST25	1995	Ascitic fluid	0.25	0.25	0.25	0.25	0.25	0.25	1.5
KP4378	ST147	1995	Blood	0.25	0.5	ND (0.25-1.0)	0.25	0.25	0.25	-
KP4387	ST158	1995	Blood	0.25	0.25	0.25	0.5	0.5	0.5	0.75
KP4389	ST25	1995	Blood	4.0 (1.0)	0.5	ND (0.25 and 0.5)	0.25	0.25	0.25	-
KP4408	ST25	1996	Blood	0.25	0.25	0.25	0.5	0.25	0.25	1.0
KP689	ST25	1999	Blood	0.25	0.25	0.25	0.25	0.25	0.25	1.0
KP725	ST12	1999	Blood	0.25	0.25	0.25	0.25	0.25	0.25	0.75
KP730	ST20	2000	Blood	ND (0.5 and 1.0)	ND (0.25-4.0)	ND (0.25 and 0.5)	0.5	0.25	0.25	-
KP748	ST14	2001	Blood	0.25	0.25	0.25	0.25	0.25	0.25	1.0
KP776	ST45	2001	Blood	0.25	0.25	0.25	0.25	0.5	0.25	1.0
KP804	Unknown	2002	Blood	0.25	0.25	0.25	0.25	0.25	0.25	1.0
KP809	ST12	2002	Blood	ND (0.25-1.0)	1.0	ND (0.25 and 0.5)	0.25	0.25	0.25	-
KP828	ST12	2002	Blood	0.25	0.25	0.25	0.25	0.25	0.25	1.0
KP829	ST12	2003	Blood	0.25	0.25	ND (0.25-8.0)	0.25	0.25	0.25	1.0
KP840	ST43	2003	Blood	1.0	1.0	2.0	0.5	0.5	0.5	0.75

Supplementary Table S1 – *continued*

Strain	ST	Year of Isolation	Environmental/ Biological source	Broth microdilution MIC (µg/mL) (Skipped wells)			Agar dilution MIC (µg/mL)			MTS MIC (µg/mL)
				Replica A	Replica B	Replica C	Replica A	Replica B	Replica C	
KP850	ST15	2003	Blood	0.25	0.25	0.25	0.25	0.25	0.25	1.0
KP874	ST15	2003	Blood	0.25	0.25	0.25	0.25	0.25	0.25	1.0
KP875	ST15	2003	Blood	0.25	0.25	ND (0.25 and 0.5)	0.5	0.5	0.5	1.0
KP888	ST15	2003	Blood	0.25	0.5	0.25	0.25	0.25	0.25	1.0
KP898	ST45	2004	Blood	ND (0.25 and 1.0)	0.25	0.25	0.25	0.25	0.25	1.0
KP910	ST15	2004	Blood	0.25	0.25	0.25	0.25	0.25	0.25	1.0
KP918	ST15	2004	Blood	1.0	1.0	1.0	0.25	0.25	0.25	1.0
KP919	ST15	2004	Blood	1.0	1.0	1.0	0.25	0.25	0.25	1.0
KP931	ST15	2004	Urine	1.0	2.0	1.0	0.25	0.25	0.25	1.0
KP972	ST15	2004	Blood	0.25	0.25	0.25	0.25	0.25	0.25	1.0
KP986	ST15	2004	Blood	1.0 (0.25)	0.25	0.25	0.25	0.25	0.25	1.0
KP997	ST15	2004	Blood	0.25	0.25	0.25	0.25	0.25	0.25	1.0
KP1000	ST15	2004	Blood	ND (0.25 and 0.5)	0.25	0.5	0.25	0.25	0.25	-
KP1001	ST15	2004	Blood	0.25	0.25	0.25	0.25	0.25	0.25	0.75
KP1003	ST11	2004	Blood	0.25	0.25	0.25	0.25	0.25	0.25	0.5
KP1019	ST15	2005	Blood	0.25	0.5	0.25	0.25	0.25	0.25	1.0
KP1025	ST15	2005	Blood	ND (0.5; 1.0 and 8.0)	2.0	ND (0.25 and 0.5	0.25	0.25	0.25	-
KP1031	ST15	2005	Blood	0.25	0.25	0.25	0.25	0.25	0.25	1.0
KP1032	ST15	2005	Blood	0.25	0.25	0.5	0.5	0.25	0.25	1.0
KP1036	ST15	2005	Blood	0.25	ND (0.5-4.0)	0.25	0.25	0.25	0.25	1.0
KP1088	ST192	2005	Blood	0.5	0.25	ND (0.25-2.0)	0.25	0.25	0.25	-
KP1122	ST15	2005	Blood	0.25	0.25	0.25	0.25	0.25	0.25	1.0

Supplementary Table S1 – *continued*

Strain	ST	Year of Isolation	Environmental/ Biological source	Broth microdilution MIC (µg/mL) (Skipped wells)			Agar dilution MIC (µg/mL)			MTS MIC (µg/mL)
				Replica A	Replica B	Replica C	Replica A	Replica B	Replica C	
KP1363	ST15	2005	Blood	0.25	0.5	0.25	0.25	0.25	0.25	1.0
KP1144	ST15	2006	Blood	0.25	ND (0.25 and 0.5)	0.25	0.25	0.25	0.5	1.0
KP1209	ST35	2006	Blood	0.25	0.25	0.25	0.25	0.25	0.25	1.0
KP1264	ST15	2007	Blood	0.25	1.0 (0.25)	0.5	0.25	0.25	0.25	-
KP1495	ST147	2007	Blood	0.25	0.25	0.25	0.25	0.25	0.25	0.75
KP1507	ST133	2007	Blood	1.0	1.0	1.0	0.25	0.25	0.25	0.75
KP1528	ST39	2007	Blood	0.25	ND (0.25 and 0.5)	0.25	0.5	0.5	0.5	1.0
KP1675	ST48	2008	Blood	0.25	0.25	0.25	0.25	0.25	0.25	1.0
KP1924	ST336	2008	Blood	2.0	2.0	1.0	0.25	0.25	0.5	1.0
KP1938	ST15	2008	Blood	1.0	1.0	1.0	0.25	0.25	0.25	0.75
KP1990	ST29	2008	Blood	1.0	4.0 (1.0)	1.0	0.25	0.25	0.25	1.0
KP2162	ST336	2008	Blood	0.25	0.25	0.25	0.25	0.25	0.25	1.0
KP2169	ST15	2008	Blood	ND (1.0 and 2.0)	8.0 (1.0)	2.0	0.25	0.25	0.25	-
KP2200	ST336	2008	Blood	1.0	1.0	1.0	0.25	0.25	0.25	1.0
KP2209	ST133	2008	Urine	0.25	1.0 (0.25)	0.25	0.25	0.25	0.25	1.0
KP2224	ST2176	2008	Blood	1.0	2.0	1.0	0.5	0.5	0.5	1.0
KP2287	ST15	2008	Blood	1.0	1.0	1.0	0.25	0.25	0.25	1.0
KP2334	ST20	2008	Blood	1.0	1.0	1.0	0.25	0.25	0.25	1.0
KP2447	ST730	2008	Blood	1.0	ND (1.0 and 2.0)	1.0	0.25	0.25	0.25	1.0
KP2454	ST231	2008	Blood	0.25	0.25	0.25	0.25	0.25	0.25	1.0
KP2463	ST218	2009	Blood	1.0	1.0	1.0	0.5	0.5	0.5	1.5
KP2476	ST13	2009	Blood	1.0	ND (1.0 and 2.0)	1.0	0.25	0.25	0.25	1.5

Supplementary Table S1 – *continued*

Strain	ST	Year of Isolation	Environmental/ Biological source	Broth microdilution MIC (µg/mL) (Skipped wells)			Agar dilution MIC (µg/mL)			MTS MIC (µg/mL)
				Replica A	Replica B	Replica C	Replica A	Replica B	Replica C	
KP2497	ST134	2009	Blood	1.0	1.0	1.0	0.25	0.5	0.25	1.0
KP2564	ST11	2009	Blood	1.0	1.0	1.0	0.25	0.25	0.25	1.0
KP2568	ST336	2009	Blood	1.0	ND (1.0 and 2.0)	1.0	0.5	0.5	0.5	1.5
KP2587	ST336	2009	Blood	1.0	1.0	1.0	0.5	0.5	0.5	1.5
KP2605	ST336	2009	Blood	1.0	1.0	1.0	0.5	0.5	0.5	1.5
KP2606	ST336	2009	Blood	1.0	1.0	1.0	0.5	0.5	0.5	1.5
KP2645	ST13	2009	Blood	1.0	1.0	1.0	0.25	0.25	0.25	1.5
KP2786	ST152	2009	Blood	2.0	1.0	1.0	0.5	0.25	0.5	1.5
KP2864	ST1801	2009	Blood	1.0	1.0	1.0	0.5	0.5	0.5	1.0
KP2895	ST726	2009	Blood	2.0	1.0	1.0	0.5	0.5	0.5	1.5
KP2948	ST14	2010	Pus	2.0	1.0	1.0	0.5	0.5	0.5	1.0
KP2958	ST76	2010	Blood	0.25	0.25	0.25	0.25	0.25	0.25	1.5
KP3000	ST231	2010	Blood	1.0	1.0	1.0	0.5	0.5	0.5	1.0
KP3046	ST14	2010	Blood	1.0	1.0	1.0	0.5	0.5	0.5	1.0
KP3185	ST15	2010	Blood	1.0	1.0	ND (2.0-8.0)	0.5	0.5	0.5	1.5
KP3270	ST348	2011	Blood	0.25	0.25	0.25	0.25	0.25	0.25	1.0
KP3292	ST15	2011	Blood	1.06 (4.0)	1.06 (2.0)	8.0	0.5	0.5	0.5	1.0
KP3323	ST11	2011	Blood	1.0	1.0	1.0	0.5	0.5	0.5	1.0
KP3396	ST39	2011	Blood	ND (2.0 and 4.0)	1.0	ND (1.0 and 2.0)	0.5	0.5	0.5	-
KP3509	ST15	2011	Urine	1.0	1.0	1.0	0.5	0.5	0.5	1.0
KP3635	ST15	2011	Blood	1.0	ND (1.0-4.0)	2.0	0.25	0.25	0.25	-
KP3660	ST70	2011	Blood	1.0	1.0	1.0	0.25	0.25	0.5	0.25
KP3715	ST405	2011	Blood	1.0	ND (1.0-8.0)	8.0 (2.0)	0.5	0.5	0.5	-

Supplementary Table S1 – *continued*

Strain	ST	Year of Isolation	Environmental/ Biological source	Broth microdilution MIC (µg/mL) (Skipped wells)			Agar dilution MIC (µg/mL)			MTS MIC (µg/mL)
				Replica A	Replica B	Replica C	Replica A	Replica B	Replica C	
KP3725	ST11	2011	Blood	1.0	4.0	1.0	0.25	0.25	0.25	1.0
KP4869	ST15	2011	Blood	1.0	1.0	ND (1.0-4.0)	0.25	0.25	0.25	1.0
KP3851	ST348	2012	Blood	2.0	1.0	2.0	0.5	0.5	0.5	1.0
KP3860	ST307	2013	Blood	0.25	0.25	1.0 (0.25)	0.25	0.25	0.25	1.0
KP4845	ST15	2013	Rectal	ND (1.0-8.0)	1.0	2.0	0.25	0.25	0.25	-
KP4852	ST960	2013	Urine	4.0	2.0	ND (1.0; 2.0 and 8.0)	0.5	0.5	0.5	-
KP4855	ST37	2013	Bronchial secreation	1.0	ND (1.0-4.0)	1.0	0.5	0.5	0.5	0.75
KP4856	ST348	2014	Rectal	1.0	1.0	1.0	0.25	0.25	0.25	0.75
KP4857	ST15	2014	Rectal	2.0	1.0	1.0	0.5	0.5	0.5	96.0
KP4859	ST423	2014	Urine	4.0	2.0	1.0	0.5	0.5	0.5	-
KP4860	ST307	2014	Rectal	1.0	2.0	2.0	0.5	0.5	0.5	1.0
KP4861	ST17	2014	Rectal	1.0	2.0	2.0	0.5	0.5	0.5	0.75
KP4862	ST348	2014	Catheter	1.0	1.0	1.0	0.5	0.5	0.5	1.0
KP4864	ST35	2015	Urine	1.0	1.0	1.0	0.5	0.5	0.5	1.5
KP4865	ST70	2015	Urine	1.0	1.0	1.0	0.25	0.25	0.25	0.5
KP4871	ST307	2015	Blood	1.0	1.0	1.0	0.5	0.5	0.5	0.75
KP4878	ST307	2015	Rectal	2.0	1.0	1.0	0.5	0.5	0.5	1.5
KP4882	ST147	2016	Rectal	2.0	2.0	1.0	0.5	0.5	0.5	0.75
KP4884	ST147	2016	Rectal	>16.0	>16.0	>16.0	0.5	0.5	0.5	1.0
KP4886	ST147	2016	Rectal	1.0	1.0	2.0	0.5	0.5	0.5	1.0
KP4887	ST147	2016	Rectal	1.0	1.0	1.0	0.5	0.5	0.5	1.0
KP4889	ST307	2017	-	>16.0	>16.0	>16.0	>16.0	>16.0	>16.0	8.0
KP5505	ST13	2019	Pus	>16.0	>16.0	>16.0	16.0	16.0	16.0	1.5

Supplementary Table S1 – *continued*

Strain	ST	Year of Isolation	Environmental/ Biological source	Broth microdilution MIC (µg/mL) (Skipped wells)			Agar dilution MIC (µg/mL)			MTS MIC (µg/mL)
				Replica A	Replica B	Replica C	Replica A	Replica B	Replica C	
KP5506	ST17	2019	Urine	>16.0	>16.0	>16.0	16.0	16.0	16.0	1.0
KP5508	ST13	2019	Urine	>16.0	>16.0	>16.0	8.0	8.0	8.0	8.0
KP5509	ST17	2019	Urine	>16.0	>16.0	>16.0	>16.0	>16.0	>16.0	>256
KP5510	ST13	2019	Blood	>16.0	>16.0	>16.0	16.0	16.0	16.0	8.0
KP5511	ST17	2019	Blood	>16.0	>16.0	>16.0	8.0	8.0	8.0	2.0
KP5513	ST13	2019	Urine	16.0	16.0	16.0	8.0	8.0	8.0	1.0
KP5514	ST13	2019	Urine	>16.0	>16.0	>16.0	>16.0	>16.0	>16.0	3.0
KP5516	ST13	2019	Pharyngeal abscess	>16.0	>16.0	>16.0	16.0	16.0	16.0	2.0
KP5520	ST13	2019	Blood	16.0	4.0	16.0	0.5	0.5	0.5	1.0

MTS - Minimum Inhibitory Concentration Test Strips; Resistant and undetermined MIC isolates are highlighted in light blue and grey, respectively.
